# Nurse champions as street-level bureaucrats: Factors which facilitate innovation, policy making, and reconstruction

**DOI:** 10.3389/fpsyg.2022.872131

**Published:** 2022-08-23

**Authors:** Daniel Sperling, Efrat Shadmi, Anat Drach-Zahavy, Shirly Luz

**Affiliations:** ^1^The Cheryl Spencer Department of Nursing, Faculty of Social Welfare and Health Sciences, University of Haifa, Haifa, Israel; ^2^Ministry of Health (Israel), Jerusalem, Israel

**Keywords:** street-level bureaucrats, nurses, network, policy, champion nurses, innovation

## Abstract

**Background:**

Nurse champions are front-line practitioners who implement innovation and reconstruct policy.

**Purpose:**

To understand through a network theory lens the factors that facilitate nurse champions’ engagement with radical projects, representing their actions as street-level bureaucrats (SLBs).

**Materials and methods:**

A personal-network survey was employed. Ninety-one nurse champions from three tertiary medical centers in Israel participated.

**Findings:**

Given high network density, high levels of advice play a bigger role in achieving high radicalness compared with lower levels advice. High network density is also related to higher radicalness when networks have high role diversity.

**Discussion:**

Using an SLB framework, the findings suggest that nurse champions best promote adoption of innovation and offer radical changes in their organizations through professional advice given by colleagues in their field network. Healthcare organizations should establish the structure and promote the development of dense and heterogeneous professional networks to realize organizations’ goals and nurses’ responsibility to their professional employees, patients, and society.

## Introduction

Among the community of nurses, nurse champions are highly professional front-line practitioners who serve as key agents in implementing innovation and reconstructing policy within the organization in which they operate (Byers, 2017; Engel et al., 2021). They are often engaged in efforts which help improve the quality of care from within the organization (Miech et al., 2018; Bonawitz et al., 2020). Nurse champions may either be nominated by managers or are self-nominated, thus their role may be formal (formally nominated and performing their formally nominated role) or informal (informally emergent or nominated for a role in a different domain from the area in which they act and promote change) (Luz et al., 2019).

A champion establishes his/her leadership through three categories of behaviors: showing enthusiasm about and confidence in the success of an innovation; network building by getting the right people involved; and persisting under adversity (Howell, 2005; Luz et al., 2019). Thus, in order for nurse champions to become influential advocates of an innovation, they have to be well-connected to people and resources of the organization (Luz et al., 2021).

While the literature conceptualizes the role and responsibilities of nurse champions from the perspective of organizational nursing and nursing management, this research regards the actions taken by nurse champions within the street-level bureaucrats (SLBs) framework. SLB is a sociological theory explaining the practices and beliefs of frontline workers in public services and the various ways in which they initiate and apply policies and organizational changes during their work (Cooper et al., 2015). In their unique and influential position, SLBs are agents of social control, thereby forming policy rather than merely implementing it (Loyens and Maesschalck, 2010).

The advantages of examining the work of nurse champions from the SLB framework is that such framework uncovers the complexities of negotiating patients and nurse colleagues as well as organizational demands that otherwise are not easily understood (Foster et al., 2017). This is especially true given that nurse champions and nurses more generally work in situations which are significantly complicated and cannot be reduced to standard procedures or formats and that their discretion promotes their self-regard which is essential in their relationships with patients and the health organization in which they are positioned (Vechter and Drach-Zahavy, 2021).

Like any other street-level bureaucrats (SLBs), nurses and nurse champions specifically are positioned at the frontline of the new public administration (Hoyle, 2014), thereby delivering government funded health services to the public. They interact directly with service recipients (patients) and enjoy substantial and decentralized discretion while executing their work as autonomous agents (Alshammari and Nalubega, 2021). They work in organizations which have heavy workloads, limited formal supervision, inadequate resources and an increasing demand for their services (Lipsky, 2010). Indeed, they frequently balance a high demand for their services with a limited supply of resources while responding to the individual needs of their patients (Hughes and Condon, 2016). Especially of interest is their conflict deriving from the obligation to provide high quality health services and their performance measurement, e.g., through accreditation processes, when there are contrasting standards (Dickson and Brindis, 2019). Among their important duties, nurses act to informally reconstruct their organizations’ policies and agendas (Newans and Siddiqui, 2021), and as such they influence the quality of life of many people (Lipsky, 1980, 2010; Cohen, 2018).

In recent years, the SLB framework has been applied to nurses working in hospitals (Hoyle, 2014; Hoyle and Grant, 2015); in community settings (Walker and Gilson, 2004; Bergen and While, 2005; Hughes and Condon, 2016; Cuthill and Johnston, 2019); and to clinical educators (Visekruna et al., 2017) or school nurses (Dickson and Brindis, 2019). However, application of the SLB framework to nurses’ roles and impact was made in anecdotal empirical studies, usually focusing on individual nurses and their behaviors as relatively stable individual characteristics.

While nurses work under formal rules, procedures and protocols, and within hierarchical organizational structures, they enjoy a considerable degree of professional autonomy with regard to their clinical practice. This discretion allows them to carefully try out new practices, ideas or behaviors, engage in creative problem-solving processes or to explore shortcuts to get things done. Furthermore, nurses, as patients’ gatekeepers, are a key part of the interdisciplinary team, advocating for patients’ needs (Bonawitz et al., 2020). Thus, the social network they create may help them in raising effective ideas, and identifying leverage opportunities for their ideas (Wichaikhum et al., 2020). Furthermore, and as suggested by the literature, organizational members use these networks to shape organizational outcomes to deliver benefits to society in a just way (also known as pubic value) (Brunetto et al., 2018). Innovativeness, including with regard to promoting nurses’ professional duties is a distinctive feature of SLBs’ work. Indeed, it is found that innovative SLBs are nominated more often as important work colleagues by their peers in workplace networks than other colleagues (Nisar and Maroulis, 2017).

## Backgroud and hypotheses development

The article takes on a theoretical approach by which nurse champions can be regarded as SLBs. Such an approach has some advantages. The SLB framework aims at exploring the complex ways by which frontline workers interact with the public to supply goods and services, and the exercise of their discretion which creates but also restricts the possibilities offered to their clients as a result of their values, economic incentives, and social networks (Cohen, 2018). The framework illuminates how, while being remote from the centers of power, SLBs’ direct interaction with their citizen clients influences policy implementation and innovation, and affects the lives and fate of many individuals (Hupe and Hill, 2007; Cohen and Hertz, 2020). This is especially true among healthcare SLBs who face additional challenges of having to balance between various growing demands for healthcare services and, at many times, being confronted with emotionally challenging situations and conflicts while working in highly stressful and intensive work environment (Brunetto et al., 2020). Innovation and creativity in the healthcare system are also required in light of rising costs and high expectations of improving care and its outcomes, maximizing convenience, access and simplicity while also aiming at spending less time and public money (Copeland et al., 2018). Their focus lies on the routines, decision-making and devices of SLBs to cope with uncertainties and work pressure that effectively become the public policies they carry out (Lipsky, 1980).

The literature emphasizes that SLBs’ interactions and relational environments affect their discretion (Lotta and Marques, 2020) and performance (Siciliano, 2017). Moreover, SLBs social relations can also promote or inhibit their innovative behavior. This is done through the characteristics of those in the SLBs’ network relating specifically to the types and amount of resources that are required to support the SLBs’ decision making and actions. Alternatively, it is facilitated through the network structure, within which SLBs are positioned in the larger organization, which as a result provides them with some advantage, such as greater diversity of innovative ideas or access to means of support required for trying out new ideas (Maroulis, 2017).

A champion’s social network comprises all the counterparts that are related to the champion, including nurses within and outside the ward, other health professionals, managers and administrators, relatives, friends, and colleagues (Luz et al., 2021). Social network theory reviews the web of connections the ego holds with other members of the network (i.e., alters) as well as the structures and relationships among the network members (Borgatti and Foster, 2003; Valente, 2010; Halgin and Borgatti, 2012; Luz et al., 2021). The network structures identify the resources that champions can access and develop because of the structural characteristics of their networks; whereas network relationships describe the resources that champions can access due to the quality of relationships with other members, who are termed “alters” (Nahapiet and Ghoshal, 1998; Ansari et al., 2018).

A prevalent, well-studied, structural characteristic of the ego’s (i.e., the champion’s) network is the network’s density, namely the level of connectedness or cohesion among alters (Borgatti et al., 2014; Ansari et al., 2018; Luz et al., 2021). High-density—specifically closed, structured, and constrained networks, favor easy interaction, communication, and cooperation between members and enables loyalty, familiarity, and shared experiences among them (Luz et al., 2021). It is possible, however, that this may come at the cost of obstructing alternative sources of influence, which is absolutely necessary for radical innovation (Yan and Guan, 2018). The strong sense of familiarity and loyalty, widespread in high-dense networks, may result in over-reliance on known information and methods of action, as well as a reluctance to advance new ones (Yan and Guan, 2018). Accordingly, radical changes that are about knowledge creation, may precisely entail low-density networks characterized by fewer constraints on network member, higher access to exclusive informational resources and risk taking (Borgatti et al., 2014; Cannella and McFadyen, 2016).

*H1: network density will be negatively related to the radicalism of the innovation.*

Yet, network density’s effect on the project’s radicalness, namely on the extent to which the project offers a significant deviation from existing products and processes, might be contingent on the varied different roles played by actors within the network. The radicalism of the project, due to its inherent uncertainty and complexity, may benefit from dense networks provided that it is multi-role diverse, namely comprised of a complex web of various health care professionals, and multi-level administrators. A role-diverse network may provide efficient and timely access to more diverse and remote information and knowledge enabling the creation of new knowledge, thereby compensating for the network’s high diversity (Yan and Guan, 2018). However, when the network is dense but less role-diverse, such as when the alters consists of a concentrated number of nurses, it is less likely that valuable knowledge and experience will be acquired or the *status quo* will be challenged, which are intrinsic features of radical change (Yan and Guan, 2018).

*H2: Network density interacts with network’s role heterophily such that the relationship between network density and project’s radicalism is less negative when role heterophily is higher.*

Furthermore, social capital is a resource involving not only structures, namely patterns of linkages, but also relationships, namely the features of social interaction associated with structures such as the norm, trust, and values attached to the ties between the ego and its alters (Mishra, 2020). In this vein, the perception of the champion that the alters provide important advice relevant to change implementation seems vital and may attenuate the negative relationship between network density and the projects’ radicalism. We propose that network density might be less harmful to the project’s radicalism when the relationship between the ego and alters are characterized as valuable due to their high-advice characteristic. Radical change necessities diverse ideas and perspectives. Thus, network density is not enough, but rather must be complemented by the ego’s perception that alters provide useful advice in terms of greater insights into the research frontier, latest trend and/or unexplored opportunities (Mishra, 2020). Conversely when network density is high but the ego does not appreciate the advice gained from his or her alters, network density might harm the project’s radicalism.

*H3: Network’s density interacts with the ego’s perception of valuable advice gained from alters such that the relationship between network density and project’s radicalism is less negative when mean advice is higher.*

While professional networking has been referred to in the literature as a motivating factor in promoting the roles and actions of SLB nurses, it was only in specific contexts that such effects have been found. For example, as a means of understanding who the key decision makers are; how decision makers may be influenced; how to identify and leverage opportunities (Benton et al., 2017); and how to promote effective communication when policy proposals are discussed (Wichaikhum et al., 2020). More generally, the way in which networks and SLBs’ relational profiles affect policy implementation, policy change and innovation has not been sufficiently analyzed in the literature (Maroulis, 2017; Loyens, 2019). No study has referred to the properties of such nurse networks or examined the specific exchanges made between network members as potential sources for policy making and reconstruction.

Moreover, very few empirical studies have examined the way nurse champions interact, their networks, the types of innovations and changes they bring about to their organizations, and the conditions that enbable them to realize their potential. If at all, and from theoretical perspectives, studies on nurse champions were made under Roger’s Diffusion of Innovations, highlighting that diffusion is the process by which an innovation is communicated over time among the participants in a social system (Luz et al., 2019). To the best of our knowledge, no empirical study has ever analyzed nurse champions’ actions and interactions as SLBs. This study therefore focused on nurse champions’ engagement with radical projects, namely projects which would likely result in a substantial change of the *status quo* (Thijssen et al., 2021). The purpose of the study was to understand the factors that facilitate or inhibit nurse champions’ engagement with radical projects, representing their actions as SLBs.

## Materials and methods

### Participants

Nurses from all hospital units of three medium-large tertiary medical centers were invited to participate in the study. They were asked to identify a present champion in their unit, based on four-stage procedure. At the first stage, the head nurse and deputy head nurse of each participating unit were asked to identify a nurse who was involved in initiating and implementing an innovative project. Second, the head nurse, the deputy head nurse, and one staff nurse were asked to name nurses who fit the following criteria: “people who (1) adopt the innovation-project as their own, and show personal commitment to it; (2) contribute to the project by generating support from other people in the organization; and (3) advocate the project beyond job requirement in a distinctive manner” (Markham, 1998, p. 491). Selected nurses were those who met the most criteria by most raters. Third, the proposed champion candidates were asked to verify that they meet the above criteria. Fourth, confirmation of the selection was made by another staff member linked to the identified project (Luz et al., 2019). Of the 128 nursing units approached, 34 either the head nurse or the champion refused to participate or could not identify a champion in their unit. Thus, the final sample included 94 nursing units (participation rate of 73%), with 94 nurses identified as champions of innovation in their unit.

### Study design

The study used a personal-network cross-sectional design with a multi-source (front-line nurses, project leaders, innovation experts) approach to data collection. The unit of analyses was the hospital’s unit. The institutional review boards of the participating hospitals (1777-14-SMC; BNZ-0082-14; RMB-0448-14) and the ethics committee of the University of Haifa (41/602) approved the study.

### Data collection

Egocentric network data were collected and recorded *via* computer-assisted, questionnaires administered to the champions by one of the authors [SL], using VennMaker 1.5.3 (Schönhuth et al., 2014) software. Specifically, a personal-network survey was used to capture the network of each primary work-related group’s contacts (alters) for one focal champion (ego; i.e., the champion). This consisted of a standardized survey employed according to the three steps recommended by Burt (1984). In Step 1, “name generators” were asked to choose alters who met the criteria in the name-generator items. Namely, the ego (champion) was asked to list individuals (alters) with whom they shared work-related issues: “From time to time, people discuss work-related issues with friends, family, colleagues, etc. In the last 6 months, with whom have you discussed work-related issues?” In Step 2, “name interpreters” were asked to answer questions about alters and the relationship between them (the ego) and their alters. We first collected sociodemographic data on alters such as ethnic identification, education, and gender. This information was used to compute network compositional and dispersion relational indices, such as the network ethnic homophily or maximum age. Second, we measured the strength of the ties between the ego and alters, using highly the standardized, highly employed questions (Perry et al., 2018). Specifically, we asked champions to describe the connections (e.g., contact frequency, tie strength, tie duration) between nominated alters (Burt, 1992; Burt et al., 2012). In Step 3, “name interrelators” were asked multi-item questions about the links between pairs of alters in order to measure the network structure, such as its density.

### Independent variables

#### Innovation type

We defined four types of innovations that best describe common innovations implemented at the unit level (Luz et al., 2019), based on the innovation types in hospital settings developed by West and Anderson (1992).

#### Human resource management

This describes innovations aimed to develop staff competence and proficiency within the unit.

#### Services

This includes all innovations designed to directly improve or expand patient services.

#### Quality control

This relates to innovations directed toward improving the efficiency, effectiveness or safety of service delivery.

#### Administration

This includes innovations in bureaucratic efficiency or those that indirectly support the core work activity.

#### Network density

This was operationalized with the question “Please reflect upon the relationships between the individuals noted above: how well do those mentioned (name 1; name 2; etc.) know each other? “not at all” or “not very well” (0), to “very well” (3).” The scale was calculated as the sum of actual responses divided by the sum of total possible responses. Thus, the higher the ratio, the greater the network density (Sparrowe et al., 2001; Crossley et al., 2015).

#### Mean advice

This was measured with the question “Please reflect upon your relationships with each of the individuals noted above: how much do you discuss clinical issues with each of them (name 1; name 2; etc.): “not at all” or “not very frequent” (1), to “very frequent” (4).” The alter-ego advice network was calculated as the mean advice the ego seeks across his or her network (Sparrowe et al., 2001; Crossley et al., 2015).

#### Role heterophily

We operationalized the ego–alter role heterophily using the E-I Index (Krackhardt and Stern, 1988). This is defined as the number of alters who hold different roles in the hospital from the ego (external ties; E) minus the number of alters who are in the same role as the ego (internal ties; I), divided by the total number of alters. Thus, a higher number indicates a greater role heterophily (Krackhardt and Stern, 1988). The index ranges from -1 (ego has ties only with alters in the same role category as the ego, showing perfect homophily) to +1 (ego has ties only with alters in different role categories from the ego, showing perfect heterophily; Crossley et al., 2015; Perry et al., 2018). To calculate the E-IIndex, wedefined similarity as belonging to the same role in the hospital as the ego, among four role groups: (1) bedside nurses, including the unit’s preceptors; (2) ward management (e.g., head of the department, head nurse, his/her deputy); (3) intermediate hospital management (e.g., supervisor, nurse coordinators, clinical specialists); and (4) senior hospital management.

#### Type of project

This captured whether the innovation aimed to improve (a) Administrative aspects, i.e., projects aimed at facilitating administrative work (e.g., organizing and summarizing hospital protocols, developing new computerized records; 20 projects); (b) Quality Control (QC): projects designed to implement or extend within-hospital QC procedures (e.g., writing and implementing protocols for treating children with trauma injuries, pain management, stroke management, assessment management; 37 projects); (c) HR: projects introduced to develop or manage HR within the unit (e.g., a program for the induction of new nursing staff, a program for team-building training; 12 projects); or (d) Service: innovations designed to directly improve or extend patient care (e.g., the development of support groups for patients and their families; patient education; assessment tools for treating nausea and vomiting for hemato-oncology children; 24 projects). This variable was derived from project descriptions as detailed elsewhere (Luz et al., 2019).

#### Initiation level

This denotes whether the innovation was top-down, stemming from higher managerial organizational levels, or bottom-up, as initiated by the champion: 0 = bottom-up; 1 = top-down.

### Dependent variables

#### Project radicalness

This factor represents champion nurses’ level of engagement in policy making or reconstruction. It was assessed due to the recommendations of Amabile (1983) and West and Anderson (1996). Classification was carried out by three domain-relevant experts, namely nurses in senior-management positions responsible for large-scale innovation-implementation processes in hospitals. They received a description of the innovation projects, were blind to the hospital of origin, and rated each project on 1-item radicalness scale that has been previously used with hospital samples (e.g., West and Anderson, 1992; Somech and Drach-Zahavy, 2013). The three experts simultaneously assessed the data and were asked to rate on a 5-point Likert-type scale “The extent to which this project would be likely to result in a substantial change to the *status quo* (1 = “not at all radical” to 5 = “extremely radical”).” In cases of disagreement, discussions were held until a consensus was reached. The projects’ radicalness scale was also dichotomized into “low-moderate” and “high” using the below and at or above the mean thresholds.

### Control variables

We controlled for network size and nurse champion seniority since previous studies have indicated that these variables can affect project outcomes (Luz et al., 2019).

### Statistical analysis

We first examined the relationship between the independent variables and the dichotomous variable of “level of radicalness” using *t*-tests and chi-square tests for continuous and categorical variables, respectively. Variables that were found to be significantly associated with low-moderate vs. high radicalness at the *p* < 0.05 level were subsequently entered into the multivariate linear regression model. In drawing the interaction plots, we followed the recommendations of Dawson (2014), with values of ±1 standard deviation serving as low and high values of the independent variable.

### Ethical considerations

The study was approved by the hospitals’ ethical review boards (1777-14-SMC; BNZ-0082-14; RMB-0448-14) and the University of Haifa’s ethics committee (41/602). Participants were asked to sign consents forms, and the confidentiality of their data was ensured. Although the study was not anonymous, each individual obtained a pseudonym and was informed that findings would be kept confidential.

## Findings

We approached 128 units in the 3 tertiary hospitals. From these, 34 units refused to participate or could not identify a champion in their unit. Three more hospital units were excluded due to missing data. Therefore, the participation rate was 71% and the final sample included 91 nursing units with 91 nurses identified as champions of innovation in their unit.

Table 1 presents the various projects according to their types.

**TABLE 1:** Projects according to types.

Type	Examples	Number of projects
Administrative (*N* = 20)	Handouts and tools for staff for improving care processes (e.g., Medication information handouts for staff, Implementing a tool for improving the shift handoff process)	5
	Electronic Health Record tools (e.g., development of a new EHR audit and feedback tool)	6
	Organization of the care environment (e.g., the organization of an infant food preparation facility, the development of a checklist for medical device count)	7
	Organization of a unit’s internal procedures protocols	2
Quality control (*N* = 34)	Preparation for audit and accreditation procedures (e.g., oversight and assimilation management of the unit’s internal operating procedure)	6
	Direct care improvement projects (e.g., the implementation of pain management and assessment tools, infection prevention management, pressure ulcer audits, and the management of prevention plans)	28
Human resources (*N* = 14)	Human resource development tools (e.g., the development of a strategic plan and oversight for prompting staff professionalism, Promoting a unit safety culture)	6
	Staff training (e.g., orientation for new nurses, staff training for removing surgical drains)	8
Service	Introduction of a completely new service (e.g., Implementing palliative care management service, New treatment modality for pain management in cancer patients)	17
	Introduction of support services (e.g., Family support group, Post bariatric surgery support groups for patients)	6

Table 2 shows the study population’s characteristics overall and according to the level of each project’s radicalness. The mean age of the nurse champions was about 40 years and the majority (∼ 80%) were female. The most common type of project was *quality control* and the least common was *human resources*. Slight differences were found among project types in terms of radicalness, yet these only trended toward statistical significance (*p* = 0.057). There was no difference between low-moderately radical projects to those rated as highly radical in terms of the network characteristics, except for role diversity (*p* = 0.044).

**TABLE 2:** Sample characteristics.

	Total	Low-moderate radicalness	High radicalness	*P*-value^‡^
	N (%)	N (%)	N (%)	
Age [mean (SD)]	39.4 (8.1)	40.5 (9.7)	38.5 (6.4)	0.232
**Gender**				
Male	17 (18.7)	6 (14.3)	11 (22.4)	0.319
Female	74 (81.3)	36 (85.7)	38 (77.6)	
**Champions’ seniority in nursing**				
Less than 15 years	51 (56.0)	22 (52.4)	29 (59.2)	0.134
Above 15 years	40 (44.0)	20 (47.6)	20 (40.8)	
**Project type**				
Administrative	20 (22.0)	13 (31.0)	7 (14.3)	0.057
Quality control	34 (37.4)	18 (42.9)	6 (32.7)	
Human resource	14 (15.4)	4 (9.5)	10 (20.4)	
Service	23 (25.3)	7 (16.7)	16 (32.7)	
**Network characteristics [mean (SD)]**				
Network density	0.59 (0.12)	0.59 (0.12)	0.58 (0.13)	0.942
Network size	11.0 (5.2)	10.4 (4.4)	11.4 (6.0)	0.364
Advice ego seeks from network	3.30 (0.56)	3.22 (0.56)	3.36 (0.54)	0.230
Role diversity	−0.38 (0.38)	−0.47 (0.28)	−0.30 (0.44)	0.044

^‡^P-values derived from t-tests for continuous variables and chi-square for categorical variables.

Linear Regression analyses revealed that none of the control variables regarding the nurse champions’ seniority and network size were significantly related to a project’s radicalness (Table 3, Model 1).

**TABLE 3:** Results of the linear regression analysis for predicting projects’ radicalness.

	Model 1		Model 2		Model 3	
	Controls		Independents and moderators		2-way interactions	
Variables	b	SE	b	SE	b	SE
Champion’s seniority in nursing	0.02	0.04	−0.06	0.05	−0.05	0.05
Network size	0.01	0.01	0.02	0.01	0.02	0.01
Networks’ density			0.38	0.52	−5.78*	2.76
Mean networks’ advice			0.15	0.13	−1.00*	0.49
Role diversity			0.55**	0.17	−0.91	0.65
Density × Mean advice					1.99*	0.82
Density × Role diversity					2.11*	0.93
F	0.274		2.81*		4.5**	
Df	2		5		7	
*R* ^2^	0.01		0.14		0.28	

SE, Standard error; Df, Degrees of freedom. *p < 0.05, **p < 0.01.

**FIGURE 1:**
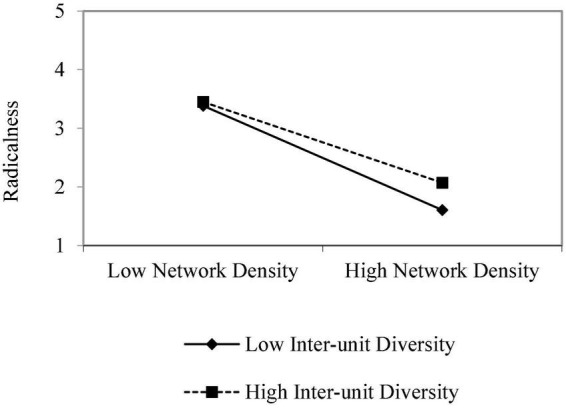
Radicalness by network density and inter-unit diversity.

**FIGURE 2:**
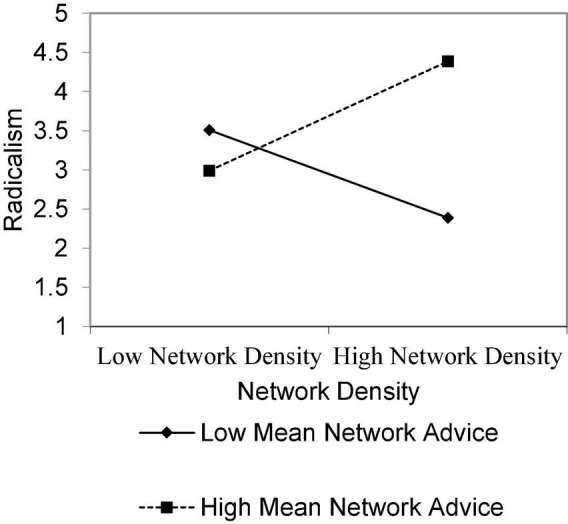
Radicalness by network density and mean advice.

The results did not support our first hypotheses that network density will be negatively related to the radicalism of the innovation. Of the independent variables, only role heterophily was significantly related to project’s radicalness (*b* = 0.55, *p* < 0.01), suggesting that the higher the role diversity among the champion and his or her network partners, the higher the project’s radicalism (Table 3, Model 2). According to our second hypotheses, network density interacts with network’s role heterophily, such that the relationship between network density and project’s radicalism is less negative when role heterophily is higher. Our results indeed show that the two-way interaction between the network density and role diversity among network partners was significant (*b* = 1.99; *p* < 0.05; Figure 1). Network density was more negatively associated with a project’s radicalism when role diversity among members was low. In regards to our third hypothesis, that network’s density interacts with the ego’s perception of valuable advice gained from alters such that the relationship between network density and project’s radicalism is less negative when mean advice is higher, the two-way interaction between the network density and mean advice the champion gains from his or her network partners was significant (*b* = 1.99; *p* < 0.05; Figure 2). Network density was positively associated with a project’s radicalism only when a nurse champion gained high advice from network partners.

## Discussion and recommendations

This study found that there are two conditions through which SLB framework helps understand how champion nurses play a key role, and are able to lead and promote radical and innovative projects, thereby applying or reconstructing policy in their field of expertise. These conditions are: (1) the champion nurses need to be part of a dense and sufficiently heterogeneous network in terms of the roles played by its members; (2) the network is characterized by an exchange of clinical advice (as opposed to mere social or interpersonal connections) between its members and SLB nurses. Significantly, the existence of one or two of these conditions is not enough. Hence, it was found that network density was more negatively associated with a project’s radicalism representing champion nurses’ actions in policy making and reconstruction when role diversity among its members was low.

With the expansion of SLB settings and occupations (Brodkin, 2012), our study highlights the role of networks in promoting policy making and reconstruction among SLBs in the nursing profession. It demonstrates that SLBs do not act alone, and that their actions are dependent on professional advice given by colleagues in their field network. While the exact mechanism that facilitates engagement in policy and innovation is yet to be explored, our findings add to current knowledge in the field of SLBs according to which dense networks in which there are closer and more constant relations, can enhance trust, thereby creating gains in communication capacity and exercise of control (Lotta and Marques, 2020).

This study emphasizes how SLB nurses’ knowledge about how others in their professional–and not only social–network think about clinical issues may result in a policy change or application (Wood and Vedlitz, 2007; Keiser, 2010). Consultations with peers in their network allow SLBs nurses to examine unofficial expectations, exercise discretionary behaviors and receive recognition of their interdependence in pursuit of their common goals. It helps them implement improvement initiatives and make sense of desired policy and organizational changes (Nisar and Maroulis, 2017). Overall, it may lead to a more robust and resilient healthcare service delivery system (Masood and Nisar, 2022). As local managers, nurse champions are critical about policies and organizational priorities, and they may not be ready to implement them. Moreover, through these actions and involvement SLBs champion nurses reconstruct and fulfill their informal accountability to the network and to their institution more broadly (LeRoux et al., 2019).

Recent publications on SLB focus more on the discretion that SLBs exercise applying the concept of negotiated discretion, that is the idea that street-level bureaucracy and street-level discretion should be understood in terms of the negotiation between bureaucrats and clients (Johannessen, 2019). The current research adds to the existing literature by highlighting an important but ignored aspect linked to nurses’ discretion, that is, nurses’ networking and its contribution to decision-making where networking serves as a platform for the exchange of clinical advice, thereby also serving as a source of creativity in the healthcare workplace (Nembhard and Lee, 2017). This aspect is significant not only because of its contribution to the practice of nurses’ clinical autonomy, but it also both reshapes the value of nurses’ discretion and facilitates their ability to exercise task discretion with their patients, families, and communities (Bonawitz et al., 2020). It follows the idea that SLBs apply and reconstruct policy making and application not through a top-down linear model, but by formal and informal networks of players and policy implementers whose actions take place beyond (or outside) the neutral space of policy delivery (Cuthill and Johnston, 2019; Houtgraaf, 2022), which is narrowly viewed as occurring only between the nurse and her patients.

In addition, nurses in this study reconstructed policy especially in the area of quality control and in the context of accreditation policy. This suggests that as SLBs, champion nurses mediate conflicting demands and the impact of policy to ensure that services are provided with due care and quality. Through the application and reconstruction of quality control cases, SLB nurses demonstrate how they use discretion to adjust, reconstruct and at times also oppose and not just respond to policy initiatives (Brodkin, 2011; Newans and Siddiqui, 2021). It emphasizes how nurses’ resistance and cooperation through heterogeneous professional networks can co-exist with regard to formal policy and how they can seek clinical advice from their network peers to mediate policy and organizational demands not only with the needs of their patients, families and communities (Hughes and Condon, 2016), but also with their colleagues’ clinical and professional understandings which shape their own values.

Theoretically, our study demonstrates how social network measures can be used to better understand SLBs’ actions in implementing, challenging or reconstructing policy, aiming to change the *status quo* and improve the shared network’s goals (Mischen and Jackson, 2008). From this perspective, it highlights the embedded nature of SLBs’ work and the impact of their interactions, thereby challenging the one-sided understanding of individual discretion and initiatives of SLBs, which does not reflect their real life practice (Loyens, 2019). Further studies, especially qualitative studies, should investigate the various mechanisms and conditions by which SLBs, and nurses in particular, maximize policy outcomes within their organization and through inter-organizational networks to redefine new goals for themselves and their organization.

Our findings acknowledge the importance of nurses as SLBs that routinely may facilitate radical innovations, thereby carrying important practical implications for policy makers who wish to promote this phenomenon. As argued in the literature, to engage in innovation, SLBs need support factors, especially effective organizational support (van den Heuvel et al., 2014; Brunetto et al., 2020). Healthcare organizations should establish the structure and spread the values for the development of dense and heterogeneous professional networks to realize organizations’ goals and responsibility to their professional employees, patients and society. This could be attained *via* establishing formal cross-ward, cross-hospital, and cross-professional forums to discuss success and failures. The participation of patients and public representatives in these forums is also imperative (Koskinas et al., 2022). Beyond the effect of such forums in enhancing learning, these forums can directly affect the diversity of nurses’ networks and prevent knowledge stickiness. In establishing these forums, managers should be attentive to the dynamic nature of networks and their evolving structural and functional characteristics. It is recommended that they adopt clear and enforceable rules and procedures to facilitate such networks and support their members in reaching collectively accepted solutions and ideas for innovation through the lead of nurse champions (De Corte et al., 2017). Yet, as our findings revealed, structural solutions in the form of diverse network are not enough. Nurses and other healthcare practitioners should combat the value that “a good professional copes by his own” and strive for mutual advice and support among teams.

## Conclusion

In their function as SLBs, nurse champions engage in radical and innovative projects, and as such they apply or reconstruct policy in their field of expertise. Our study shows that to maximize their role in this context, nurse champions need to be part of a dense and sufficiently heterogeneous network in terms of the roles played by its members. Moreover, such a network is characterized by an exchange of clinical advice between its members and SLB nurses, thereby enhancing nurses’ professional competency and resulting in better outcomes for the nursing community and the organization within which they take part of.

## Data availability statement

The original contributions presented in this study are included in the article/supplementary material, further inquiries can be directed to the corresponding author.

## Ethics statement

The institutional review boards of the participating hospitals (1777-14-SMC; BNZ-0082-14; and RMB-0448-14) and the Ethics Committee of the University of Haifa (41/602) approved the study. The patients/participants provided their written informed consent to participate in this study.

## Author contributions

DS: conceptualization, writing—original draft preparation, writing—review and editing, and project administration. ES and AD-Z: conceptualization, methodology, writing—original draft preparation, writing—review and editing, and supervision. SL: methodology and investigation. All authors contributed to the article and approved the submitted version.
